# Functional Restoring in Parsonage-Turner Syndrome With a Multimodal Rehabilitation Program: A Case Report

**DOI:** 10.7759/cureus.80383

**Published:** 2025-03-11

**Authors:** Eric Chun-Pu Chu

**Affiliations:** 1 Chiropractic and Physiotherapy Centre, New York Medical Group, Hong Kong, CHN

**Keywords:** brachial neuritis, chiropractic, neuralgic amyotrophy, parsonage-turner syndrome, scapular winging

## Abstract

Parsonage-Turner syndrome (PTS) is a rare disorder characterized by sudden unilateral shoulder pain followed by progressive neuralgic amyotrophy. This case report describes an effective multimodal therapy approach for a 63-year-old man who presented with severe right shoulder pain and right scapular winging, which are uncommon manifestations of PTS. The patient participated in a multidisciplinary treatment program that included spinal manipulation, physiotherapy, magnetic muscle stimulation, and scraping therapy, all customized specifically for his distinct symptoms and underlying neuromuscular issues. Shoulder stability, range of motion, and pain relief all improved significantly after an eight-week treatment. This study demonstrates the effectiveness of a multimodal rehabilitation strategy in treating such complex conditions and adds to the limited literature on the management of PTS with neuralgic amyotrophy. It stresses the significance of a comprehensive therapy approach, utilizing multiple therapeutic modalities to restore patient neuromuscular function. Further investigation is needed to corroborate these results through controlled studies.

## Introduction

Parsonage-Turner syndrome (PTS), also known as brachial neuritis, is a rare disease and is characterized by acute unilateral shoulder pain and progressive neuralgic amyotrophy [1]. The syndrome is caused by acute neuritis of the brachial plexus. PTS can occur spontaneously or be provoked by infections, surgery, or physical trauma. The annual incidence of PTS is estimated at about two to three individuals per 100,000 [2], with men being affected more commonly than women and typically in their middle age. Symptoms typically begin with debilitating shoulder pain, which gradually subsides over several weeks, followed by variable degrees of muscle weakening and neurological disturbances. The diagnosis is mostly clinical, supplemented by imaging studies and electrodiagnostic tests used to rule out other causes of similar symptoms.

Scapula winging (scapula alata) is a rare yet debilitating condition. It occurs when the muscles surrounding the scapula are either too weak or paralyzed to keep it stable. As a result, either the medial or lateral edge of the scapula protrudes from the back, forming wings. This debilitating condition can hinder the ability to push, pull, and lift large items, in addition to performing daily tasks like carrying shopping bags and brushing teeth and hair [3]. The long thoracic nerve is derived from the superior trunk of the brachial plexus and is the motor nerve for the serratus anterior muscle, which pulls the scapula forward around the thorax. PTS often results in a winged scapula; however, other peripheral nerves can also be affected [4].

PTS is a challenging and often overlooked clinical condition, as illustrated in the case given here of a 63-year-old man six months after a direct strike to his right shoulder, a known but less common trigger. Additionally, the patient's delayed onset of shoulder pain and the presence of a supraspinatus tendon tear confounded the clinical picture, masking the underlying brachial neuritis. This example demonstrates the complexity of identifying PTS when there are concomitant orthopedic issues and underscores the necessity of evaluating PTS in individuals experiencing unexplained shoulder pain and muscle weakness after trauma. The effective management of this situation through a combination of physiotherapy, chiropractic manipulation, and innovative therapeutic modalities serves as a valuable reference for other similar cases in clinical practice, illustrating the potential for significant recovery even in cases with late diagnosis.

## Case presentation

A 63-year-old man with left-handed dominance presented to the chiropractic clinic with a chief complaint of severe right shoulder pain and right scapular winging. His past medical history was unremarkable for neurological or autoimmune disorders, and he reported no known allergies or family history of neuromuscular conditions. He was a recreational tennis player with no previous shoulder injuries and maintained moderate physical activity levels. He had no recent illness, vaccination, or surgery that might trigger an autoimmune response.

Six months prior to presentation, he fell and hit his right shoulder directly into a wall while renovating his home. Following the impact, he experienced mild to moderate neck and right shoulder pain that he self-managed with over-the-counter non-steroidal anti-inflammatory drugs (NSAIDs). Approximately two days after the initial trauma, while playing tennis, he experienced sudden weakness and a distinctive cracking sound in his right shoulder while hitting a ball, followed by moderate shoulder pain that persisted after the game. The patient specifically noted that despite the pain and weakness, there was no loss of sensation in the right upper limb at any point.

**Figure 1:**
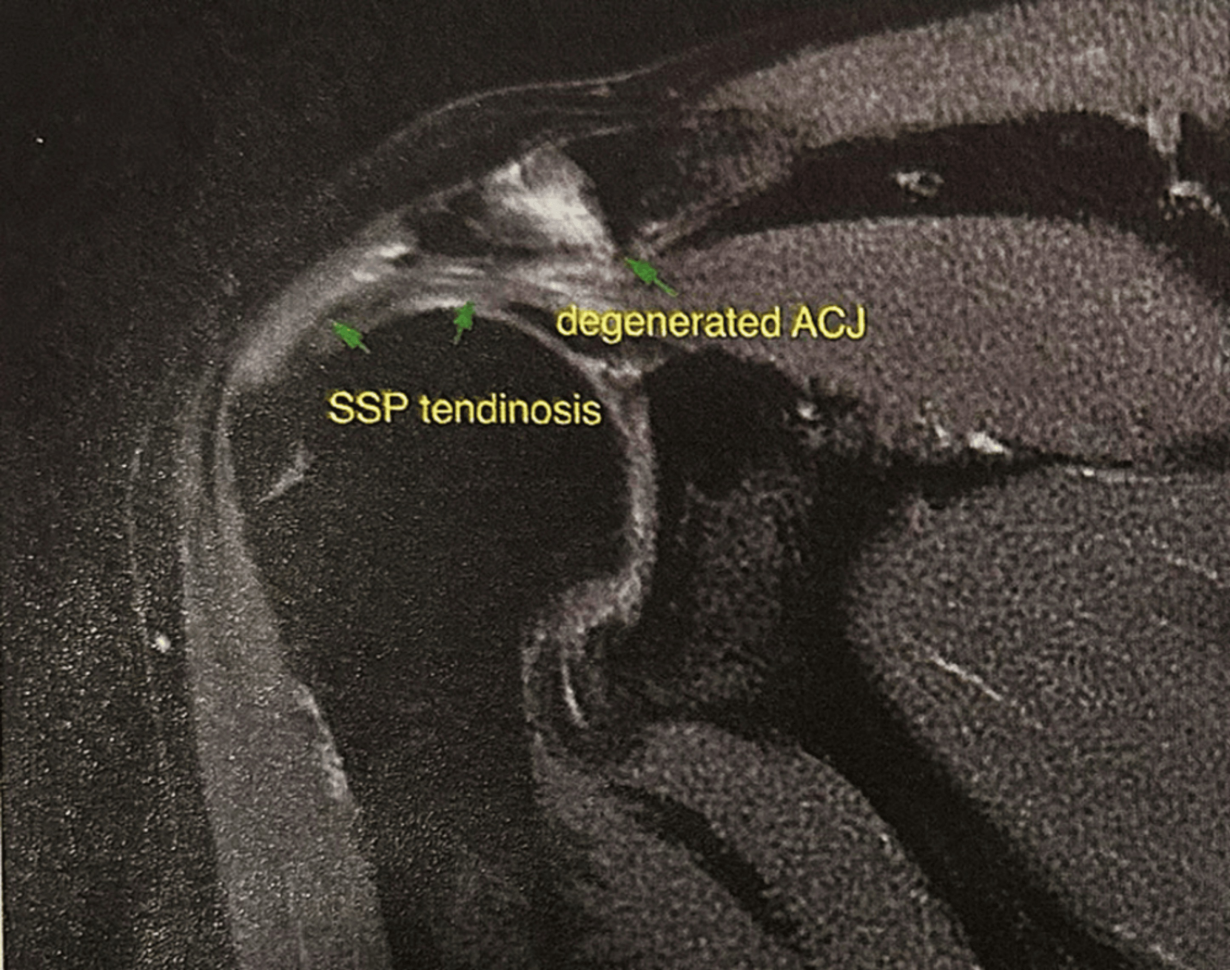
Coronal T2 fat sat magnetic resonance imaging (MRI) Showing a fluid-filled subacromial-subdeltoid (SASD) bursa with partial tearing of the supraspinatus (SSP) tendon at its insertion (green arrows). ACJ: acromioclavicular joint

Concerned about the persistent symptoms, he initially sought care from his orthopedic surgeon three weeks after the injury. Diagnostic imaging, including an MRI, revealed a partial tear of the right supraspinatus tendon (Figure 1). Based on this finding, his condition was diagnosed and treated as a mechanical supraspinatus tendon tear. The prescribed treatment consisted of physical therapy three times weekly for eight weeks and a progressive strength training program focusing on rotator cuff rehabilitation. Despite adhering to the treatment regimen, his weakness persisted with minimal improvement, and he began to notice a visible abnormality in his right scapular position.

For the two days immediately preceding his chiropractic consultation, the patient reported an acute exacerbation with progressive weakness throughout the right upper limb and severe pain in the right shoulder radiating to the upper arm, which interrupted his sleep pattern. He rated his pain as a "5" on a numerical rating scale (NRS 0-10; 0: no pain; 10: worst pain imaginable). He had attempted to manage the increased pain with over-the-counter analgesics (acetaminophen 1000 mg every six hours), which did not provide adequate relief. The patient also observed pronounced right scapular winging, particularly evident when attempting to raise his arm forward, accompanied by significant weakness in external rotation and abduction of the right shoulder. He reported finding it impossible to lift his right arm above shoulder level and described difficulty with daily activities such as combing his hair, reaching overhead cabinets, and driving. Having failed to improve with conventional orthopedic management, he sought chiropractic treatment, specifically for shoulder rehabilitation and functional recovery.

Examination revealed that the patient exhibited normal cervical lordosis. A comprehensive neurological and musculoskeletal assessment was performed, including cervical spine evaluation, which showed a full range of cervical motion without pain. The Spurling test and other neurological provocative tests of the neck were negative. Mild tenderness was palpable over the right supraclavicular fossa; however, there were no signs of abnormal swelling. The range of motion in the right shoulder was full and free of pain. The patient had normal sensory function, deep tendon reflexes, and motor function in the right upper extremity, except for shoulder girdle muscles.

**Figure 2:**
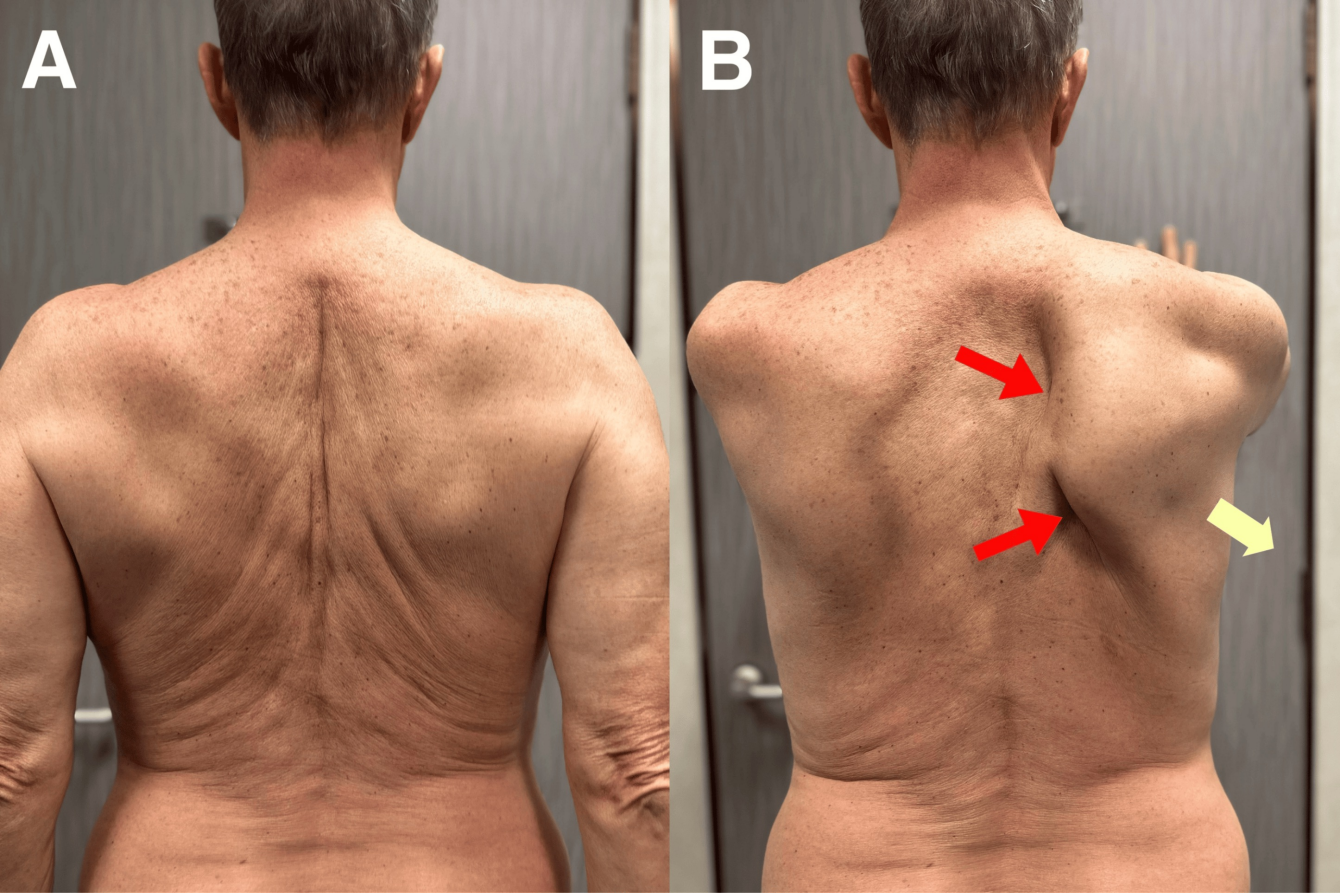
Medial scapular wing due to serratus anterior weakness A: The altered position of the scapula can be slight in the resting position of the arms; B: but medical scapular winging (red arrows) and absent pull of serratus anterior (yellow arrow) are evident when the patient performs forward flexion of the arms.

During forward shoulder flexion, the right medial scapular border was notably displaced from the thoracic cage, indicating medial scapular winging (Figure 2). There was slight hypotrophy of the right deltoid, right biceps, right supraspinatus, and right infraspinatus muscles. Manual muscle testing was performed using a standardized protocol, with particular attention to the muscles innervated by the various branches of the brachial plexus. Infraspinatus manual testing [5] to assess force mainly exerted by the infraspinatus muscle showed mild weakness of the right forearm against the examiner's resistance, rated as 4 out of 5 with a dynamometer. The serratus anterior muscle strength was significantly reduced (2/5).

The differential diagnosis included cervical radiculopathy, rotator cuff pathology, disuse hypotrophy of shoulder girdle muscles, brachial plexopathy, and post-traumatic isolated long thoracic nerve injury (post-traumatic scapulae alata). Given the history of direct trauma to the shoulder, post-traumatic nerve injury was a significant consideration. To further differentiate between these conditions, he was referred for a radiological assessment. His EOS® (EOS Imaging, Paris, France) full-body radiographs revealed mid-thoracic scoliosis and degenerative spinal disease.

To explore the possibility of brachial plexopathy versus isolated nerve injury, the patient was referred for a detailed electrodiagnostic evaluation. Electromyography (EMG) and nerve conduction studies (NCS) showed slightly prolonged latency of the right serratus anterior muscle along with preserved compound muscle action potential (cMAP) amplitudes. Additionally, careful needle examination revealed not only denervation potentials in the serratus anterior but also subtle changes in the supraspinatus and infraspinatus muscles, suggesting a broader involvement of the brachial plexus rather than an isolated long thoracic nerve injury. These findings were consistent with chronic motor axonal degeneration attributable to neuralgic amyotrophy rather than a simple traumatic mononeuropathy.

Based on the clinical presentation of initial pain followed by weakness in multiple muscle groups, the distribution of muscle involvement beyond a single peripheral nerve territory, and the supportive electrodiagnostic findings, a diagnosis of Parsonage-Turner syndrome (brachial neuritis or neuralgic amyotrophy) was made, despite the confounding traumatic event.

The treatment strategy was customized to regain muscular function and encourage the growth of muscle tissue. The multimodal rehabilitation program included physiotherapeutic exercises designed to enhance the strength and flexibility of the shoulder girdle, spinal manipulation, and joint mobilization techniques to achieve proper alignment of the thoracic spine and shoulder structures, magnetic muscle stimulation to activate and strengthen the underlying musculatures [6], and scraping therapy (Gua Sha) [7] to improve the functionality performance of the immune, nervous, and soft tissues, thus enabling effective therapeutic results. The multimodal rehabilitation program included physiotherapeutic exercises designed to enhance the strength and flexibility of the shoulder girdle. Spinal manipulation was performed using a diversified technique with high-velocity, low-amplitude (HVLA) thrusts targeting the T3-T7 vertebral segments to address the vertebral function and improve thoracic mechanics. Joint mobilization techniques included grade III-IV Maitland mobilizations (2 Hz frequency, three-minute duration) applied to the glenohumeral and scapulothoracic articulations, specifically targeting posterior capsular restrictions and facilitating proper scapulohumeral rhythm.

Magnetic muscle stimulation was administered with a figure-of-eight coil, delivering repetitive pulses at the serratus anterior (along the mid-axillary line at the level of ribs 5-7), supraspinatus (superior to the scapular spine), and infraspinatus muscles (infrascapular fossa) with gradually increasing intensity from 20% to 60% of maximum stimulator output over the treatment period, totaling 20 minutes per session. Instrument-assisted soft tissue mobilization (Gua Sha) was applied using a stainless-steel tool with medium pressure on the upper trapezius, levator scapulae, and rhomboids for five minutes per muscle group.

**Figure 3:**
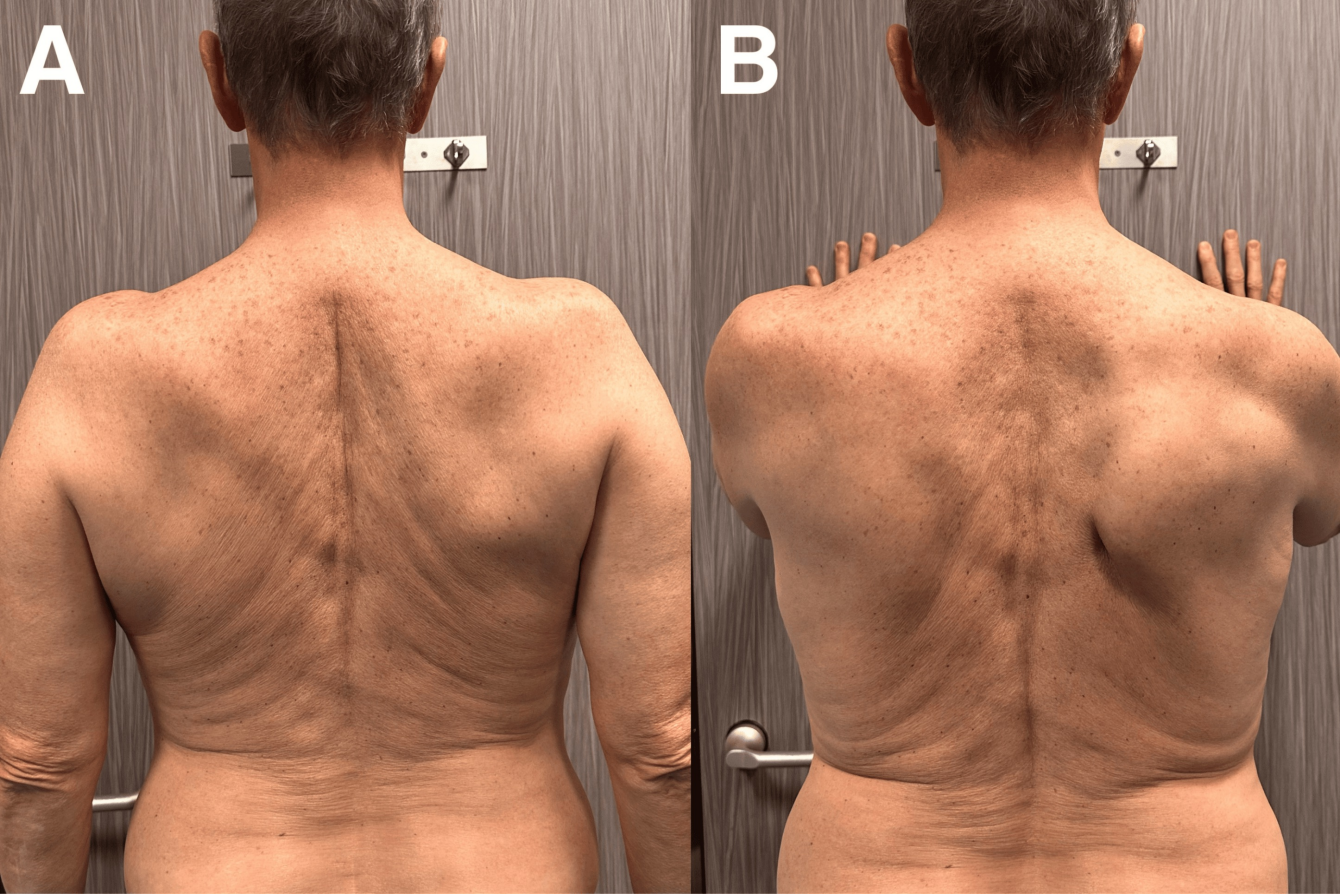
Monitoring of the same patient An eight-week multimodal rehabilitation program leads to complete pain relief and a satisfactory functional recovery of the upper limb, even though muscle hypotrophy is not completely regained when compared visually to the initial photographs. A: position at rest; B: forward flexion of the arms

Initially, the patient participated in three sessions each week during the first four weeks. The patient indicated that his muscle strength had improved by 50%, and he could lift his arms. The patient's shoulder pain score dropped from 5 to 2 on a numeric pain rating scale. Following this, the number of sessions was reduced to twice a week for the subsequent four weeks. While clinical appointments continued, a home program that included wall push-ups, scapular push-ups, scapular retractions, and chest stretches was implemented, concentrating on strengthening the serratus muscle and improving shoulder stability. Furthermore, guidance on appropriate mechanics during activities and information on injury prevention were given. Patient adherence to the home-based program was assessed using a self-reported exercise diary, which revealed a compliance rate of 62% with the prescribed exercises. After an eight-week course of treatment, all complaints had completely disappeared, allowing the patient to participate fully in daily and recreational activities, although muscle loss was not entirely recovered when evaluated through visual comparison to the earlier photographs (Figure 3). The patient was advised to return afterward for a monthly evaluation to maintain improved results and to monitor therapeutic progress. No adverse effects associated with the treatment were observed in the patient. On the fourth month re-evaluation, the patient's motor strength returned to normal, and he reported complete recovery.

## Discussion

Parsonage-Turner syndrome (PTS), commonly referred to as brachial neuritis, is a rare and progressive form of neuralgic amyotrophy characterized by sudden unilateral shoulder pain, succeeded by progressive neuralgic amyotrophy [1]. The pathophysiology of PTS, although not entirely understood, is believed to encompass a combination of immune-mediated and mechanical factors that result in nerve inflammation and injury. The most common explanation involves an autoimmune response in which the body’s immune system incorrectly attacks the brachial plexus nerve fibers, causing inflammation and subsequent neural injury [8]. This autoimmune response may be triggered by infections, surgical procedures, vaccinations, or trauma, which might clarify the abrupt onset of symptoms following such events [9]. The brachial plexus comprises a network of nerves that is derived from the lower cervical spine (C5, C6, C7, and C8) to the shoulder, arm, and hand. The inflammatory process of PTS primarily targets motor neurons, leading to muscle weakness and progressive neuralgic amyotrophy, while sensory neurons are less frequently affected, which may be responsible for the preservation of sensation despite significant motor deficits [10].

**Figure 4:**
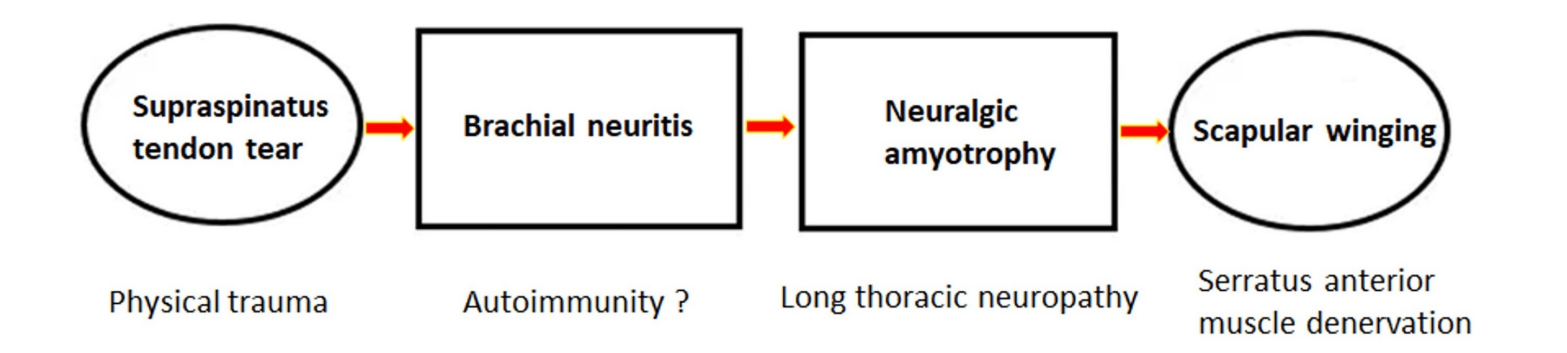
Progression of Parsonage-Turner syndrome in a 63-year-old man Flow diagram illustrating the connection between underlying neuromuscular problems and clinical features.

The term “scapular winging” has been used interchangeably with scapular dyskinesis. However, when scapular motion is impaired by a neurological disorder, the term “scapular winging” is more appropriate. As seen in this case, the serratus anterior muscle has been denervated due to long thoracic nerve injury and is too weak to stabilize the scapula, causing medial scapular winging (Figures 2, 4). Neurogenic scapular winging occurs when a portion of the scapula moves excessively away from contact with the rib cage immediately after commencing arm motion and remains so throughout all stages of arm movement [11]. This debilitating condition can make it difficult to pull, push, and lift heavy objects, as well as to do daily activities such as carrying grocery bags and brushing teeth [3].

PTS can be difficult to diagnose because of its inconsistent presentation and symptoms that are similar to those of other neuromuscular disorders. The rarity of PTS makes diagnosis even more challenging due to a lack of clinical suspicion. It is possible to confuse the initial severe pain with a more prevalent ailment like cervical radiculopathy, rotator cuff injuries, or other brachial plexopathies when it is followed by muscle weakness and atrophy. Electromyography (EMG) and nerve conduction studies (NCS) are essential diagnostic tools that help differentiate PTS from other disorders by identifying the specific nerves affected and the extent of the damage. Following an injury to the innervating nerve, muscle fibers begin to discharge spontaneously. It is advisable to conduct EMG four to six weeks after the onset of symptoms [12]. If EMG is conducted earlier, denervation potentials (positive sharp waves and fibrillation potentials) indicative of axonal injury may go undetected. Months later, there is a reduction in positive sharp waves and fibrillation potentials [13]. In this context, the timing of ordering an EMG concerning symptom onset may impact diagnosis, treatment, and prognosis.

There is no known cure, but conservative treatment, which includes the use of analgesics, glucocorticoids, and physiotherapeutic exercises, usually brings satisfactory results. Surgical intervention involving neurolysis and transfers of tendons or nerves to enhance functional recovery is a viable option when no improvement is seen by five months [14]. Because of the intricacy of the injury, which included a supraspinatus tendon tear, long thoracic nerve injury, and neuralgic amyotrophy of the serratus anterior muscle, the current case necessitated a comprehensive diagnostic approach and a more extensive treatment regimen. The proposed multimodal treatment, taking into account the patient’s features, resulted in pain relief and a good functional recovery of the upper limb after an eight-week program, although muscle wasting was not fully restored.

This case is of significant scientific value as it demonstrates the effectiveness of a multimodal therapeutic strategy in managing a complex presentation of PTS. This case contributes to the limited literature on this rare issue and highlights the significance of an integrated treatment plan that includes multiple diagnostic modalities and rehabilitation approaches. However, it is important to acknowledge potential limitations such as the lack of a control group, reliance on patient-reported outcomes, and the likelihood of bias in therapy administration and documentation. These factors may influence the generalizability of the results and suggest that further controlled studies are needed to better understand the full effects and mechanisms of manual interventions in similarly complex clinical presentations.

## Conclusions

Parsonage-Turner syndrome is rare and is often underdiagnosed by physicians because of insufficient clinical suspicion. This report illustrates a comprehensive assessment of a patient who presented with unilateral shoulder pain and scapular winging, along with a multimodal therapy approach to address the underlying neuromuscular issues contributing to confounding clinical features. A multimodal conservative strategy is effective for treating this unusual condition.
